# Spatial-Light-Modulator-Based Multichannel Data Transmission by Vortex Beams of Various Orders

**DOI:** 10.3390/s21092988

**Published:** 2021-04-24

**Authors:** Svetlana N. Khonina, Sergey V. Karpeev, Muhammad A. Butt

**Affiliations:** 1 IPSI RAS-Branch of the FSRC “Crystallography and Photonics” RAS, 443001 Samara, Russia; khonina@ipsiras.ru (S.N.K.); karp@ipsiras.ru (S.V.K.); 2 Department of Technical Cybernetics, Samara National Research University, 443086 Samara, Russia; 3 Institute of Microelectronics and Optoelectronics, Warsaw University of Technology, Koszykowa 75, 00-662 Warszawa, Poland

**Keywords:** Vortex beams, multiplexed signal, data transmission, multichannel diffractive optical element, demultiplexing, sorting, spatial light modulators

## Abstract

We report an atmospheric multichannel data transmission system with channel separation by vortex beams of various orders, including half-integer values. For the demultiplexing of the communication channels, a multichannel diffractive optical element (DOE) is proposed, being matched with the used vortex beams. The considered approach may be realized without digital processing of the output images, but only based on the numbers of informative diffraction orders, similar to sorting. The system is implemented based on two spatial light modulators (SLMs), one of which forms a multiplexed signal on the transmitting side, and the other implements a multichannel DOE for separating the vortex beams on the receiving side. The stability of the communication channel to atmospheric interference and the crosstalk between the channels are investigated.

## 1. Introduction

The use of vortex beams for communication in free space [1,2,3,4,5] and optical fibers [6,7,8,9,10] provides several advantages in terms of noise immunity and allows multichannel communication to be organized [11,12]. Signals from different channels are transmitted by vortex beams of different orders. Multiplexing is achieved by traditional optics elements, while demultiplexing is accomplished by both diffractive optical elements (DOEs) matched with beams in various channels [11] and special elements ensuring beam sorting [10,13,14,15]. These methods have certain difficulties in implementation, due to both a large number of elements and the complexity of their alignment and the need for two-phase elements with specific functions.

Laser beams with a vortex phase dependence of the form exp(imφ), named optical vortices (OVs), where *m* is the order of the optical vortex beams, which is also called topological charge (TC), have the orbital angular momentum (OAM) proportional to *m*. Vortex beams with integer values *m* are orthogonal, which is used for (de)multiplexing communication channels.

The method of beam recognition that is based on a multichannel DOE [16], which can be used both for the recognition of vortex beams [17,18] and arbitrary polarization-phase distributions [19,20,21], has a very good potential for mode division multiplexing (MDM) techniques. The idea of recognizing vortex beams using multichannel DOEs was proposed for the first time in works that were devoted to measuring the OAM [22,23]. Subsequently, these DOEs were used in the problems of fiber-optic multichannel communication utilizing vortex beams [24,25]. More recent studies [26,27,28,29] used spatial light modulators (SLMs) to implement previously proposed DOEs.

In implementing a method that is based on the optical correlation of incident radiation and modes recorded in a multichannel DOE, it is necessary to measure correlation peaks, the intensities at the centers of diffraction orders, which is achieved either using photodetector matrices [30,31] or by placing an optical fiber at the center of the diffraction order [32]. The second method seems preferable because it does not require mathematical processing of the measured intensity matrices and, therefore, it can be used for high-speed data transmission.

This paper discusses ways of realizing DOEs for recognizing vortex beams not only integer, but also half-integer orders. At the same time, in contrast to previous works that are based on the use of multichannel DOEs, an approach is considered without digital processing of the output images but only based on taking the numbers of informative diffraction orders into account. This approach is similar to sorting and it can be implemented using fiber optics devices. The results also include an experiment on the transmission of a multiplexed signal formed by an SLM in the atmosphere with its further demultiplexing using the proposed DOE.

## 2. Development of Multichannel Filters and Modelling of the Sorting Process

MDM is based on maintaining the orthogonality of optical modes when passing through optical media and systems [33,34,35,36,37,38,39], which provides a simple way to demultiplex information that corresponds to different mode channels based on matched filters [16,28,40,41]. MDM-based information encoding in a physical medium is easily accomplished using diffractive optics. Let us consider a field whose complex transmission function is a linear superposition of mode functions $\Psi_{n}\;(x,y)$ with given weights in the Cartesian coordinates:

(1) $$w\left(x,y\right)=\sum_{n=1}^{N}c_{n}\Psi_{n}\left(x,y\right)$$

The light field that is defined by Equation (1) is a multidimensional optical signal, the information which is varied by the coefficient $c_{n}$.

To decode this information, it is convenient to use multichannel filters that are matched by a set of complex conjugate modes in different diffraction orders. The transmission function of the filter is defined, as follows:

(2) $$\tau (x,y)=\sum_{p=1}^{P}\Psi_{p}^{*}\left(x,y\right)\exp\left[i\left(\alpha_{p}x+\beta_{p}y\right)\right],$$

where α*_p_* and β*_p_* are the carrier frequencies. The light field distribution after the filter in the focal plane defined by the Cartesian coordinates (*u*, *v*) is described by the Fourier transform [18]:

(3) $$F(u,v)=\frac{k}{f}\int_{-\infty }^{\infty }\int_{-\infty }^{\infty }w(x,y)\tau (x,y)\exp\left[-i\frac{2\pi }{\lambda f}\left(ux+vy\right)\right]dxdy\approx \frac{k}{f}\sum_{p=1}^{P}c_{p}\delta \left(u-\frac{\lambda f}{2\pi }\alpha_{p},v-\frac{\lambda f}{2\pi }\beta_{p}\right),$$

where $\delta \;(u-u_{p},v-v_{p})$ are the shifted delta-functions, *k* = 2π/λ is the wavenumber, λ is the wavelength of laser radiation, and *f* is the focal length of the used lens.

Thus, the intensity at given points of the focal plane, corresponding to the centers of diffraction orders

(4) $$\left(u_{p},v_{p}\right)=\left(\frac{\lambda f}{2\pi }\alpha_{p},\frac{\lambda f}{2\pi }\beta_{p}\right)$$

will be approximately proportional to the squares of the moduli of the coefficients $|\;c_{n}{|}^{\;2}$ in Equation (1).

The basis of optical vortices is the most convenient for communication channel multiplexing. In this case, the mode function is defined, as follows:

(5) $$\Psi_{m}\left(x,y\right)=\exp\left(im\varphi \right)=\exp\left(im\tan^{-1}\left(y/x\right)\right),$$

where *m* is an integer value of a topological charge (TC).

The convenience of OVs lies in the independence of the field radius, which provides versatility [22,23,24,42] and significant tolerances for aligning the optical system [17,18].

Note that functions shown in Equation (5) are orthogonal for integer TC values. However, OVs with fractional TC are also actively considered [43,44,45,46]. Moreover, the superposition of OVs with an integer TC:

(6) $$w\left(\varphi \right)=\sum_{n=1}^{N}b_{n}\exp(im_{n}\varphi )$$

generally, has a non-integer TC.

Because the TC of the light field can change during propagation and passage through optical systems [47], it is more convenient to consider a conserved characteristic of the field, such as the OAM [48]. A field with a fractional TC [exp (i μφ)], like superposition defined by Equation (6), can be expanded based on integer OVs that are defined by Equation (5). Subsequently, OAM can be calculated by the following formula [22,23,40]:

(7) $$\mu =\left(\sum_{m=-\infty }^{\infty }m{\left|b_{m}\right|}^{2}\right){\left(\sum_{m=-\infty }^{\infty }{\left|b_{m}\right|}^{2}\right)}^{-1}$$

In practice, use is made of the final version of Equation (7):

(8) $$\mu_{M}=\left(\sum_{m=-M}^{M}m{\left|b_{m}\right|}^{2}\right){\left(\sum_{m=-M}^{M}{\left|b_{m}\right|}^{2}\right)}^{-1}$$

Algorithms are also known that allow one to calculate the field OAM by three [49] and even two [50] expansion coefficients. However, in all cases, it is necessary to accurately measure the relative values of these coefficients. This is unacceptable in communication systems transmitting information in real-time; therefore, OV sorting, rather than measuring, is usually considered, i.e., binary detection—presence (1) or absence (0) of a signal in a certain channel. In the case of matched multichannel DOEs, this corresponds to the presence or absence of intensity at the center of a certain diffraction order, which can be detected in real-time using a system of appropriately connected optical fibers [32]. Let us consider a phase multichannel filter matched to both integer and half-integer OVs:

(9) $$\tau_{s}(x,y)=\exp\left[i\arg\left\{\sum_{p=-P}^{P}\exp\left(-i\frac{p}{2}\varphi \right)\exp\left[i\left(\alpha_{p}x+\beta_{p}y\right)\right]\right\}\right]$$

**Figure 1:**
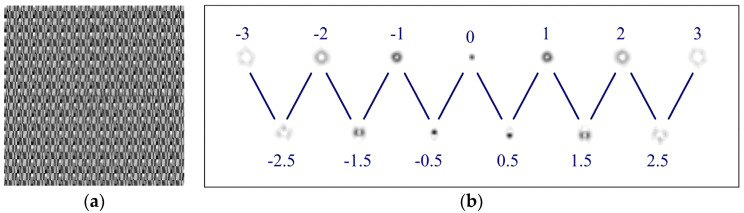
View of a 13-channel filter defined by Equation (9) at *p* = 6: (**a**) phase of the filter, (**b**) intensity pattern (negative) in the focal plane and correspondence of diffraction orders to OV values.

Figure 1 shows the view of a multichannel filter defined by Equation (9) at *p* = 6 and the correspondence of the diffraction orders to the OV values.

The binary (presence/absence) sorting of integer OVs is easily achieved using the top row of the filter that is shown in Figure 1. In this case, you just need to determine the noise cutoff threshold, for example, considering the experimentally determined crosstalk (see Section 3). Similarly, one can determine the presence/absence of half-integer OVs from the bottom row, which are orthogonal for beams with half-integer TCs.

Additional information an approximate OAM value can be obtained using the arithmetic mean formula for OV numbers with ‘pronounced’ correlation peaks:

(10) $$\mu_{A}=\frac{1}{N_{\Omega }}\sum_{p\in \Omega }m_{p}$$

where Ω is the set of diffraction orders with a central intensity above the threshold and $N_{\Omega }$ is the number of such orders. Note that Equation (10) can be applied both separately only to the upper ($\mu_{A}^{1}$) or lower row ($\mu_{A}^{2}$) and while taking all diffraction orders ($\mu_{A}$) into account. Table 1 shows the simulation results for input OVs beams with different TCs, including fractional ones, and the characteristics that were obtained from Equation (10).

As can be seen from the results shown in Table 1, the proposed filter allows one not only to easily sort integer and half-integer orders, but also quite accurately determine the fractional OAM value (for example,$\mu_{A}=-1.25$) as the arithmetic mean for all orders with ‘pronounced’ (i.e., above a threshold value) correlation peaks. Table 2 shows similar simulation results for different OV beam superpositions. Note that, in this case, a more accurate value for the OAM is obtained by applying Equation (1) to the first row ($\mu_{A}^{1}$). This is because Equation (10), for the case $\mu_{A}^{1}$, is equivalent to Equation (8) with the same weights $b_{m}$ in superposition defined by Equation (6).

## 3. Experimental Results

**Figure 2:**
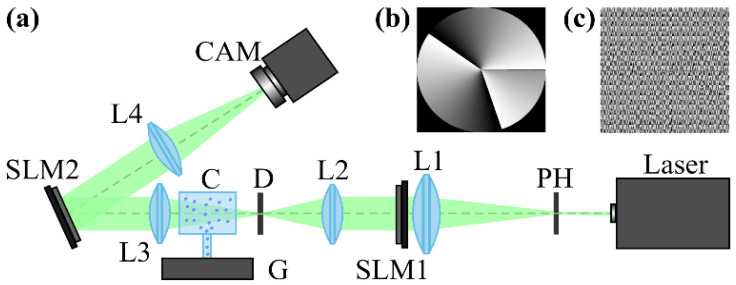
(**a**) The experimental setup for investigation of demultiplexing of OV beams and their superpositions using the proposed diffractive multichannel filter: Laser is as solid-state laser (λ = 532 nm), PH is a pinhole (aperture size 40 μm). L1, L2, L3, and L4 are lenses with focal lengths of 350, 200, 300, and 250 mm, SLM1 is a transmissive spatial light modulator (HOLOEYE, LC 2012 with 1024 × 768-pixel resolution), SLM 2 is a reflective spatial light modulator (HOLOEYE, PLUTO_VIS with 1920 × 1080-pixel resolution), D is a diaphragm, G is a vapor generator connected with a glass container, CAM is a video camera. (**b**) An example of the phase mask of the element generating the sent OAM state defined as *w*(φ) = exp (*i*2.5φ), μ = 2.5 and displayed onto the first SLM. (**c**) The phase mask of the proposed diffractive multichannel filter displayed onto the second SLM.

**Figure 3:**
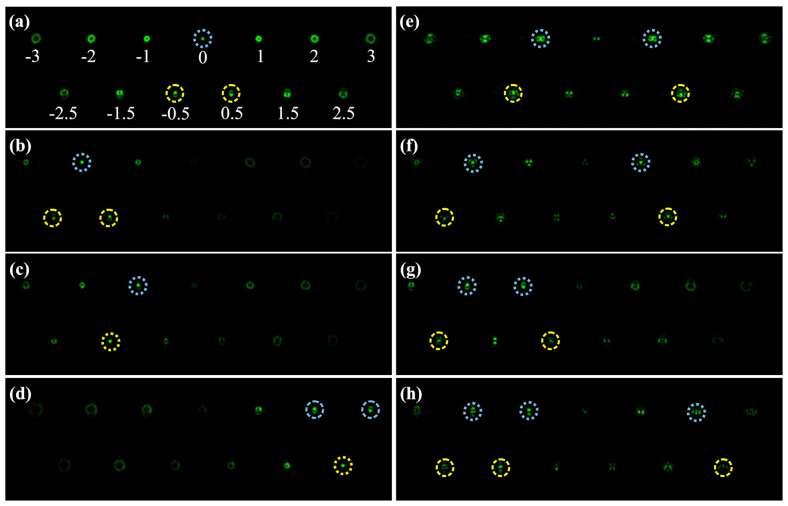
Experimentally obtained results of the detection of light fields with different OAM states: (**a**) *w*(φ) = const, μ = 0 (correspondence of the diffraction orders in the focal plane to the OAM state is shown), (**b**) *w*(φ) = exp(−*i*2φ), μ = −2, (**c**) *w*(φ) = exp(−*i*1.25φ), μ = −1.25, (**d**) *w*(φ) = exp(*i*2.5φ), μ = 2.5, (**e**) *w*(φ) = exp(−*i*φ) + exp(*i*φ), μ = 0, (**f**) *w*(φ) = exp(−*i*2φ) + exp(*i*φ), μ = −0.5, (**g**) *w*(φ) = exp(–*i*φ) + exp(−*i*2φ), μ = −1.5, and (**h**) *w*(φ) = exp(–*i*φ) + exp(−*i2*φ) + exp(*i*2φ), μ = −0.33. The color dashed circles show the generated correlation peaks.

Figure 2 shows the optical setup designed for the experimental investigation of demultiplexing of OV beams and their superpositions using the proposed diffractive multichannel filter. The input laser beam from a solid-state laser (Laser) (λ = 532 nm) was extended and spatially filtered with a system that was composed of a pinhole PH (aperture size of 40 μm) and a lens L1 (focal length of 350 mm). The collimated laser beam was directed onto the display of a transmissive spatial light modulator (SLM1) (HOLOEYE, LC 2012 with 1024 × 768-pixel resolution). This SLM was used to realize the phase masks of the input light field defining the sent OAM-states (see an example of the used phase mask in Figure 2b), and it can be considered to be an OAM-multiplexer. A diaphragm D blocked the zero-diffraction order, and a combination of lenses L2 and L3 with focal lengths of 200 and 300 mm imaged a plane that was conjugated to the plane of the first SLM display onto the display of the second reflective SLM2 (HOLOEYE, PLUTO_VIS with 1920 × 1080 pixel resolution). The second SLM was used to realize the phase masks of the proposed diffractive multichannel filter performing demultiplexing of the initial light field that is shown in Figure 2c. A lens L4 with a focal length of 250 mm focused the reflected laser beam onto the sensor of a CMOS-video camera. Figure 3 shows the example of focal plane intensity distributions generated in the cases of laser beams with different OAM-states. The experimentally obtained results are in good agreement with the modelled ones.

**Figure 4:**
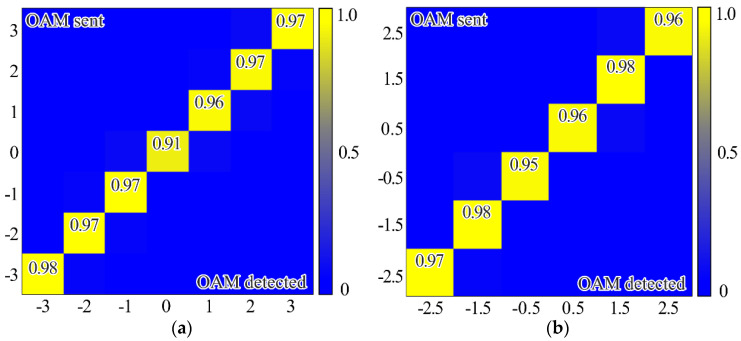
Experimentally measured crosstalk matrix of OAM states using only (**a**) integer orders (i.e., the upper row of the proposed filter shown in Figure 1), (**b**) half-integer orders (i.e., a bottom row of the proposed filter shown in Figure 1).

It is important to evaluate the crosstalk performance of the proposed diffractive multichannel filters, because the mode crosstalk can be used to determine the maximum number of OAM states that can be simultaneously transmitted without requiring space-time coding [51]. Figure 4 shows the experimental arrangement for measuring the crosstalk performance of the proposed multichannel diffractive multichannel filter using just integer or half-integer TCs. The peak value occurs when the sent and detected OAM are the same, with all off-diagonal elements having significantly lower values. In this case, the threshold level may be based on the noise level.

**Figure 5:**
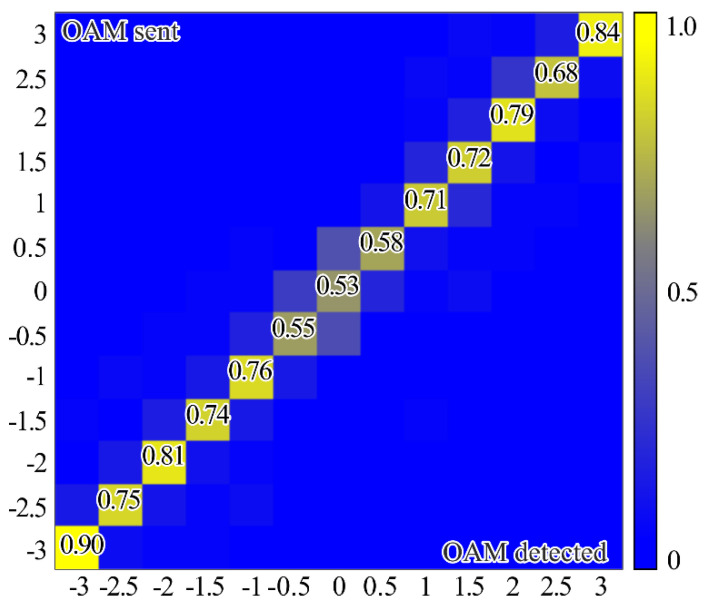
Experimentally measured crosstalk matrix of OAM states using both integer and half-integer TCs (i.e., both rows of the proposed filter shown in Figure 1).

Figure 5 shows the experimental results using both integer and half-integer TCs. These measurements are taken for each OAM-state separately and then repeated for both integer and fractional TCs of OAM-beams. In this case, as expected, the maximum energy correlation occurs when the OAM state of the sent and detected light field are the same. However, the simultaneous use of integer and fractional OAM states leads to lower peak values—the worst value is approximately 0.53, while in the case of detection only integer or fractional OAM states this value is larger than 0.9. It can be explained by the fact that, in the case of the simultaneous detection of integer and fractional OAM states using the proposed diffractive multichannel filter, correlation peaks appear not only in the diffraction orders exactly corresponding to the detected integer/fractional OAM state, but also in the neighboring diffraction orders corresponding to the fractional/integer OAM state as was mentioned in the modeling. Although, in the case of mixed integer-fractional OAM states, the efficiency of detection is less; however, it is also possible since the worst value is more than the total energy of other OAM-states. In this case, the cut-off threshold should be higher. However, note that, due to the significant difference in signal levels in the centers of different diffraction orders, the SNR in the “responded” channels will be much higher than in the other channels. It was shown in [18] that a level of 0.5 from the maximum certainly ensures the cutoff of uninformative noise, even in the case of significant turbulent distortions.

In the further experiments, for the investigation of the stability of the proposed method for the propagation of information channels through a scattering medium, we used a vapor generator that was connected with a glass container. Using a vapor generator, we created, inside the reservoir, a medium with refractive index irregularities. The average diameter of the generated aerosols did not exceed 5 μm; the spraying capacity was not less than 0.4 mL/min. The tested laser beams carried the desired configuration OAM states were going through the glass container. The continuous vapor generation resulted in changing parameters of the scattering medium inside the glass container. The scattering and refraction of light from individual vapor droplets led to distortions of the beam structure and corresponding scintillations of the correlation peaks that formed in the focal plane of the diffractive multichannel filter. The use of the so-called scintillation index is the most common method of analyzing the stability of the detection of the received OAM states in these conditions. The scintillation index is a key metric for free-space optical communications—it measures the normalized intensity variance that is caused by atmospheric turbulence. This parameter is defined, as follows [52]:

(11) $$\eta =\frac{\langle I^{2}\rangle }{{\langle I\rangle }^{2}}-1$$

where 〈⋅〉 is an averaging operation and *I* is the total beam energy in the region of interest. The bigger scintillation index values, the more difficult it is to detect the corresponding OAM state. The scintillation index measured in the case of detection of the OV beam with the TC of −2 (see Figure 2b) for 10 min. was 0.00096 and, in the case of detection of the superposition of OV beams with TCs of −1 and −2 (see Figure 2b) for 10 min., was 0.04802. It can be explained by the fact that, in the second case, two correlation peaks are formed, which leads to a redistribution of the intensity between them and an increase in the fluctuation of the intensity of each correlation peak. However, it should be noted that the value obtained in the case of detection of a single OAM state is much less than the previously obtained value of the scintillation index for the case of OV beams with TCs up to five propagating through the scattering medium and detected without any diffractive multichannel filter [17]. Besides, the value of the scintillation index that is obtained in the case of detection of the superposition of OV beams with different OAM states is comparable to these values. It can be explained by the fact that in the case of the use of the proposed diffractive multichannel filter the correlation peaks have a larger intensity density, which is confirmed by the experimentally measured cross-talk matrix of OAM shown in Figure 4 and Figure 5. Thus, the use of the filter allows one to increase the detection capability of the received OAM states in free-space communication systems through a scattering medium.

Pay attention, we only investigated the processes of multiplexing and demultiplexing in a multichannel system at certain values of signals in the transmission channels, which is, as if at fixed times. The temporal characteristics of this system have not been investigated, since they are entirely determined by the dynamic temporal characteristics of the SLM. Note that SLMs are not intended for high-speed modulation. The implementation of high-speed OVs transmission through the atmosphere in near-IR using commercially available devices have been carried out in the work [32].

## 4. Conclusions

We have proposed and implemented diffraction multichannel filters for the convenient sorting of beams with integer and half-integer TC values, which also make it possible to accurately determine the fractional OAM value. These filters allow for one to organize MDM-based multichannel communication.

The proposed demultiplexing procedure, in contrast to previous works based on the use of multichannel DOEs, can be implemented without digital processing of the output images, only based on the use of fiber optics devices. All of the operations shown in Table 1 and Table 2 have corresponding analogue implementation. In particular, the integration of the intensity in the central part of the diffraction orders can be carried out with optical fiber with a corresponding core diameter. The selection of informative orders is easily carried out by a threshold, and the ordering scheme is similar to the sorting method. For averaging the numbers of informative orders following Equation (10), an appropriate sensor can be designed.

A prototype of an optical system has been experimentally implemented, which performs multiplexing and demultiplexing of a communication channel using OV beams with various integer and half-integer TCs.

The system includes two SLMs and a vapor generator to simulate atmospheric noise. The experimentally measured crosstalk values for orthogonal OV bases are generally of the order of 0.96–0.98, which indicates a high selectivity of communication channels, even in interference conditions. The obtained results can serve as a basis for the development of a multichannel communication system, including a high-speed one, using optical fibers in the near-IR range. The experiments on the transmission of OVs were carried out for a low-mode fiber in the visible range in [7]. The optical fiber should also support the required number of modes to transmit OVs in the near-IR range.

Other advantages of the proposed configuration are as follows: resistance to atmospheric interference, which is shown by a low cross-flow and scintillation index, the simplicity of the optical scheme and the elements used in comparison with the sorting methods.

The prospects of the work are associated, first, with the choice of orthogonal sets of vortex beams with integer and half-integer TC values with the further implementation of the corresponding filters, which is very important for encrypting communication channels.

## Figures and Tables

**Table 1:** Simulation results for OV beams with different TCs μ.

Characteristics of the Input Light Field	Intensity Distributions in the Focal Plane
w(φ)=const, μ=0,	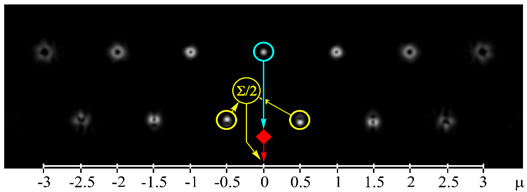
$\mu_{A}^{1}=0$, $\mu_{A}^{2}=0$, $\mu_{A}=0$	
w(φ)=exp (−i2φ), μ=−2, $\mu_{A}^{1}=-2$, $\mu_{A}^{2}=-2$, $\mu_{A}=-2$	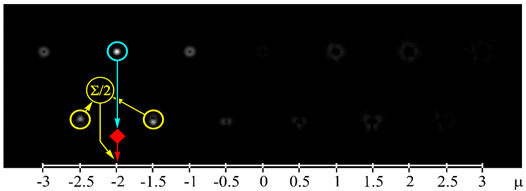
w(φ)=exp (−i 1.25 φ), μ=−1.25 $\mu_{A}^{1}=-1$, $\mu_{A}^{2}=-1.5$, 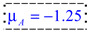	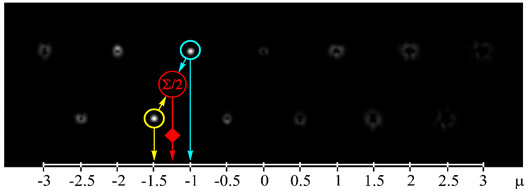
w(φ)=exp (i 2.5 φ) μ=2.5 $\mu_{A}^{1}=2.5$, $\mu_{A}^{2}=2.5$, $\mu_{A}=2.5$	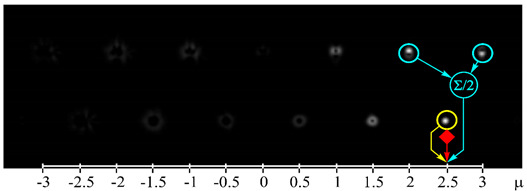

**Table 2:** Simulation results for various OV beam superpositions.

Characteristics of the Input Light Field	Intensity Distributions in the Focal Plane
w(φ)=exp(−iφ)+exp(iφ), μ=0, $\mu_{A}^{1}=0$, $\mu_{A}^{2}=0$, $\mu_{A}=0$	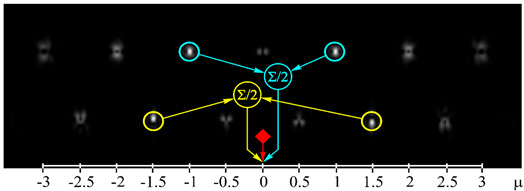
w(φ)=exp(−i2φ)+exp(iφ), μ=−0.5, $\mu_{A}^{1}=-0.5$, $\mu_{A}^{2}=-0.5$,$\mu_{A}=-0.5$	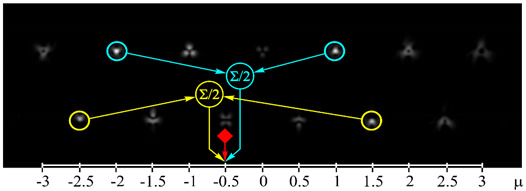
w(φ)=exp(−iφ)+exp(−i2φ), μ=−1.5, $\mu_{A}^{1}=-1.5$, $\mu_{A}^{2}=-1.5$, $\mu_{A}=-1.5$	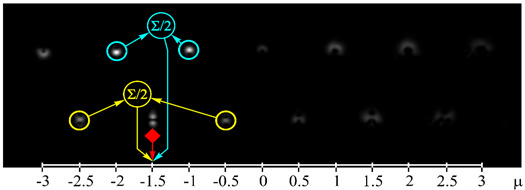
$\begin{array}{l} w(\varphi )=\exp(-i\varphi )+\\ +\exp(-i2\varphi )+\exp(i2\varphi ) \end{array}$ μ=−0.33, 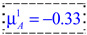 , $\mu_{A}^{2}=-0.5$, $\mu_{A}=-0.415$	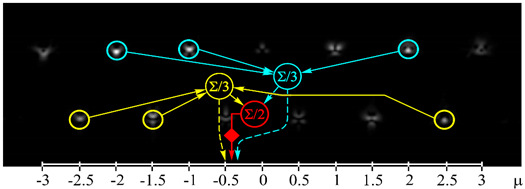

## Data Availability

Not applicable.
